# Perioperative outcomes following open radical cystectomy in the setting of locally advanced bladder cancer

**DOI:** 10.1007/s11255-026-05127-y

**Published:** 2026-04-09

**Authors:** Pierluigi Russo, Nazario Foschi, Nicolò Lentini, Francesco Pio Bizzarri, Giuseppe Maioriello, Mauro Ragonese, Chiara Ciccarese, Roberto Iacovelli, Giuseppe Palermo, Marco Campetella, Carlo Gandi, Domenico Nigro, Francesco Rossi, Domenico Varacalli, Savio Domenico Pandolfo, Or Schubert, Filippo Gavi, Daniele Fettucciari, Maria Chiara Sighinolfi, Marco Racioppi, Emilio Sacco, Bernardo Rocco

**Affiliations:** 1 Department of Urology, Fondazione Policlinico Universitario Agostino Gemelli, Rome, Italy; 2 Department of Urology, Ospedale Isola Tiberina—Gemelli Isola, Rome, Italy; 3 Section of Hygiene, Department of Life Sciences and Public Health, Università Cattolica del Sacro Cuore, Rome, Italy; 4 Department of Oncology, Fondazione Policlinico Universitario Agostino Gemelli, Rome, Italy; 5 Department of Medicine and Translational Surgery, Università Cattolica del Sacro Cuore, Rome, Italy; 6 Department of Urology, Addenbrooke’s Hospital, Cambridge University Hospitals NHS Foundation Trust, Cambridge, UK; 7 Department of Life Science, Health, and Health Professions, University Unilink, Rome, Italy; 8 Department of Urology, University of L’Aquila, 67100 L’Aquila, Italy; 9 Department of Neurosciences and Reproductive Sciences and Odontostomatology, University of Naples “Federico II”, 80131 Naples, Italy

**Keywords:** Urothelial cancer, Radical cystectomy, Advcanced Bladder cancer, Muscle Invasive Bladder Cancer

## Abstract

**Purpose:**

To compare the perioperative outcomes and long-term survivors between localized (organ-confined cT2N0M0) and locally advanced (cT3–4 and/or cN1–3,M0) bladder cancer in patients undergoing radical cystectomy and lymphadenectomy.

**Methods:**

We conducted a retrospective observational cohort study including 364 consecutive patients who underwent open RC with urinary diversion at a high-volume tertiary referral center between July 2016 and November 2024. Patients were stratified according to pathological stage into localized disease (pT0–T2N0) and locally advanced disease (pT3–T4 and/or N1). Perioperative outcomes were evaluated using multivariable regression models. Overall survival (OS) was analyzed with Cox regression and Kaplan–Meier estimates. Recurrence-free survival (RFS) and cancer-specific survival (CSS) were assessed using Fine–Gray competing-risks models. Propensity score matching (PSM) was performed as a sensitivity analysis.

**Results:**

Among 364 patients, 203 had localized and 161 had locally advanced BCa. Locally advanced disease was associated with greater intraoperative blood loss (β 230 ml, 95% CI 89.9–370.3) and longer operative time (β 16.5 min, 95% CI 2.6–30.3), but postoperative complication rates were similar between groups. With a median follow-up of 54.9 months in localized and 36.4 months in locally advanced BCa, locally advanced disease was associated with significantly worse oncologic outcomes. Multivariable analyses confirmed higher risks of overall mortality (HR 3.40, 95% CI 2.40–4.81), recurrence (sHR 5.12, 95% CI 3.45–7.59), and cancer-specific mortality (sHR 2.26, 95% CI 1.43–3.59). Results were consistent after PSM.

**Conclusion:**

This study showed that, compared with localized disease, RC for locally advanced BCa was associated with similar 30-day mortality and major complication rates, but worse oncological outcomes. These findings support the perioperative feasibility of surgery in selected patients with locally advanced disease while underscoring their persistently poorer cancer prognosis.

## Introduction

The global burden of bladder cancer is substantial, with 573,000 new cases diagnosed in 2020. Men are disproportionately affected, accounting for more than three-quarters of all cases, due in large part to their historically higher smoking rates or to genomic pathways [1]. As a result, bladder cancer ranks as the sixth most prevalent neoplasm in men and the tenth most widespread cancer worldwide [2]. The increase in bladder cancer will have a significant impact on the healthcare system as there will be a need for more expensive systemic treatments, the need for diagnostic procedures, and more admissions associated with radical cystectomy (RC).

Cisplatin-based neoadjuvant chemotherapy (NAC) followed by an RC with pelvic lymph node dissection is the favored treatment strategy for muscle-invasive bladder cancer (MIBC) patients. The management of patients with locally advanced BCa and positive lymph nodes (N +) remains unclear. Remarkably, there is a correlation between long-term survival and pN status pre-RC, reflected in 5–year overall survival (OS) rates: 81.4% for pN0, 29.3% for pN1, 18.2% for pN2, and 0% for pN3 [3]. This pattern is mirrored in the count of pN + post-RC, where the 5-year OS rates are 32% for 1–2 positive Nodes, 15% for 3–10 positive nodes, and drop to 3% for cases with more than 10 positive nodes [4]. However, a review documented that surgery and chemotherapy in N + patients compared to chemotherapy alone led to an improvement in OS (Hazard ratio 0.60, *P* < 0.001), although on multivariate analysis, this difference did not reach significance ( Hazard ratio = 0.91, *P* = 0.628), [5]. According to other studies, the preoperative mortality rate, irrespective of tumor stage, has been estimated to fall within a range of 2–5% [6], with a greater increase in this rate in older patients [7, 8].

Nevertheless, surgical interventions for advanced pelvic malignancies are associated with increased treatment-induced mortality and morbidity [9].

Also, it is crucial to emphasize that radical cystectomy is a complex operation prone to complications during and after surgery, which prevents approximately 30% of patients from receiving postoperative adjuvant therapy [10, 11]. Recent findings from other major oncological surgeries indicate a negative impact of these complications on long-term survival outcomes. It’s hypothesized that these issues might induce immunosuppression and other immune alterations, which could encourage early cancer recurrence and negatively affect long-term survival [12–14].

Despite increasing interest in Rc for locally advanced BCa, contemporary evidence remains limited regarding its perioperative safety and oncological benefit compared with RC for localized disease. Clarifying these outcomes is essential to inform patient selection, guide multidisciplinary counseling, and support the design of future prospective studies.In this study, we aimed to evaluate the perioperative safety, short-term complications, and long-term survivors of performing open RC for localized and locally advanced BCa in a propensity-matched analysis.

## Methods

### Patients and study design

This study is an observational retrospective cohort analysis that examined all individuals who underwent open RC and urinary diversion for BCa at Fondazione Agostino Policlinico Gemelli, Rome, between July 2016 and November 2024. The study has been approved by the Ethics Committee (ID 2882, approved by the EC on 05/12/2019).

Patients who underwent robot-assisted radical cystectomy (RARC) with either intracorporeal urinary diversion (UD) or extracorporeal UD via mini-laparotomy were excluded from the analysis.

Eligible patients were identified from our institutional cystectomy and muscle-invasive bladder cancer (MIBC) database and included all individuals diagnosed with MIBC or high-risk non–muscle–invasive bladder cancer (NMIBC), with a clinical or pathological T stage ranging from T1 to T4, N0–N1, or M0–M1. Three hundred sixty-four consecutive patients met the inclusion criteria and were included in the study.

All procedures were conducted by skilled surgeons with extensive experience in bladder cancer management. Radical cystectomy was performed in conjunction with hysterectomy and bilateral oophorectomy in female patients and prostatectomy in male patients as part of the standard surgical plan. Computed tomography (CT) scans of the thorax and abdomen were performed to assess lymph node involvement and distant metastases.

In our clinical practice, we prioritize neoadjuvant chemotherapy over adjuvant chemotherapy whenever feasible. This is due to evidence suggesting better outcomes with the neoadjuvant approach [15] [16]. Additionally, a significant reason for the limited use of adjuvant chemotherapy in our cohort is the occurrence of perioperative and postoperative complications following RC and lymphadenectomy. These complications can adversely affect patients’ overall health status, rendering them unfit for subsequent adjuvant chemotherapy.

Patients were stratified according to the pathological T stage, localized (T0—T2 and N0) and locally advanced (T3 -T4 and/or N1). Even Patients who did not respond to neoadjuvant chemotherapy are still considered for RC.

Standardized follow-up care after surgery adhered to local and international guidelines, incorporating regular CT scanning of the thorax and abdomen to monitor patient outcomes [17]. The factors considered to influence the outcome were patient age, sex, body mass index (BMI, kg/m^2^), smoking status, Diabetes Mellitus, neoadjuvant chemotherapy, Charlson Comorbidity index (CCI), American Society of Anesthesiologists (ASA) classification score, preoperative estimated glomerular filtration rate (eGFR) with the MDRD formula, hypertension, congestive heart failure, abdominal surgeries, prior radiotherapy and urinary derivations.

In our center, all Patients undergoing surgical procedures have followed the ERAS protocol since 2016. Specifically, during the pre-hospitalization phase, which takes places approximately 3–4 weeks prior to RC, Patients receive a clinical nutrition consultation (Nutricatt protocol) with a personalized diet and oral branched-chain amino acid (BCAA) supplementation 14 days before bladder surgery [18].

During the surgical procedure, all patients were observed and managed using a standardized approach. This included electrocardiography, pulse oximetry, end-tidal CO2, bispectral index, invasive arterial pressure, and urine output measurements. Anesthesia was induced using a combination of propofol, remifentanil, and rocuronium, and was maintained with inhaled sevoflurane. Furthermore, all patients underwent protective mechanical ventilation and had a central venous catheter inserted.

Throughout the surgery, several key physiological parameters were collected, including serum lactate concentration, PCO2 gap, venous oxygen saturation (SvO2), oxygen extraction ratio (O2ER), and the percentage of in-target time for stroke volume (SV) and mean arterial pressure (MAP).

We have previously demonstrated the critical role of maintaining MAP above a safety threshold to potentially reduce postoperative complications in major abdominal surgery [19]. Furthermore, the early administration of norepinephrine was employed to avoid prolonged hypotension, which could translate into sustained organ perfusion. These comprehensive monitoring and management strategies are aimed at optimizing patient outcomes by ensuring appropriate hemodynamic stability during the surgery. All Patients receive an extended VTE prophylaxis for 30 days.

The postoperative events of interest included mortality, readmissions, reoperations, minor and major complications, urologic-specific complications within 30 days, operating time, and length of hospital stay. All complications occurring within 90 days of surgery were classified and graded using the Clavien Dindo system.

Then, we evaluated Recurrence-Free Survival (RFS), Cancer-Specific Survival (CSS), and Overall Survival (OS) in the two groups. RFS was measured from the time of RC to the occurrence of a local recurrence or distant metastasis. CSS and OS were assessed from the point of RC to the time of death due to cancer and death from any cause, respectively.

### Statistical analysis

Descriptive statistics were utilized to summarize the data. Continuous variables were described using the mean and standard deviation or the median and interquartile range (IQR) (first and third quartiles, Q1-Q3), depending on the data distribution. Categorical variables were presented as absolute numbers and percentages. For comparison between localized and advanced bladder cancer of continuous variables, either the independent t-test or the Mann–Whitney U-test was used based on the data distribution. Categorical variables were compared using the Chi-squared test or Fisher's exact test, as appropriate. Missing data were evaluated for all study variables. Given the limited proportion of missingness and the retrospective nature of the dataset, a complete-case analysis was performed. No multiple imputation was applied.

Associations between disease stage (localized vs locally advanced) and perioperative outcomes were evaluated using multivariable regression models. Logistic regression was applied for binary outcomes and linear regression for continuous outcomes, adjusting for age, sex, BMI, GFR, neoadjuvant chemotherapy, diabetes mellitus, and ASA score. Results are presented as odds ratios (ORs) or beta coefficients with corresponding 95% confidence intervals (CIs).

OS was analyzed using Cox proportional hazards regression models and compared between groups using Kaplan–Meier curves and the log-rank test. RFS and CSS were analyzed using Fine-Gray competing-risks regression models to account for death from other causes as a competing event, with group comparisons performed using cumulative incidence functions and Gray’s test. Results are reported as hazard ratios (HRs) for Cox models and subdistribution hazard ratios (sHRs) for Fine-Gray models, with corresponding 95% confidence intervals (CIs). Median follow-up time was estimated using the reverse Kaplan–Meier method.

To further assess the robustness of the results, propensity score matching (PSM) was performed. Although disease stage is not a modifiable exposure, PSM was employed to adjust for differences in non-oncologic clinical variables that might confound peri-outcome comparisons. Propensity scores were computed using a logistic regression model based on covariates such as age, sex, BMI, GFR, neoadjuvant chemotherapy, diabetes mellitus and ASA. PSM was performed using a 1:1 ratio with a nearest-neighbor algorithm, a caliper with of 0.2 standard deviations of the logit of the propensity score, and without replacement. The balance of covariates between groups was assessed using p-values and the standardized mean difference (SMD)[20]. An absolute SMD value of less than 0.1 was preferred to indicate a good matching balance [21].

In the matched cohort, perioperative outcomes were analyzed using logistic and linear regression models, while survival outcomes were compared using Kaplan–Meier curves and the log-rank test, with additional Cox proportional hazards and Fine-Gray competing-risks regression analyses performed as appropriate.

A two-sided p-value < 0.05 was considered statistically significant for all analyses.

All statistical analyses used R version 4.4.0 (2024–04-24) for Windows. The propensity score matching analysis was carried out using the MatchIt package. We adhered to the published guidelines for reporting studies with propensity score matching [22].

## Results

### Patient Population

Of 364 consecutive patients undergoing radical cystectomy and lymphadenectomy, 203 patients had localized bladder cancer, and 161 patients had locally advanced bladder cancer. Patients with localized BCa had a higher median BMI, 26.6 (IQR 23.8–29.4) vs. 25.4 (IQR 23.3–28.3) kg/m2, p = 0.014, and were more likely to have received neoadjuvant chemotherapy (40.7% vs 28.0%; p = 0.010) and prior radiotherapy (7.4% vs 2.5%; p = 0.037). Conversely, patients with locally advanced BCa were more likely to have an ASA score ≥ 3 (29.4% vs. 46.0%; p = 0.001) and exhibited lower median GFR values, 71.0 (IQR 56.0–89.5) vs. 63.0 (IQR 44.0–83.0) ml/min/1.73m2, p = 0.001.

After propensity score matching analysis, 140 patients in the localized BCa group were matched to 140 patients in the locally advanced BCa. Following matching, all covariates were comparable between the two BCa groups (Table 1), with all SMD < 0.1 (Supplementary Fig. 1). Exceptions were urinary derivations and prior radiotherapy, the latter showing an SMD of -0.413. However, prior radiotherapy was a rare event in the cohort (n = 19; 5.2%). Urinary derivations was slight unbalanced for ileal conduit and ureterocutaneostomy, with SMDs of -0.203 and 0.248, respectively.

**Table 1:** Comparison between baseline characteristics between groups in unmatched and matched populations

median (q1-q3) / n (%)	Overall (N = 364)	Localized (N = 203)	Advanced (N = 161)	p-value	Localized (N = 140)	Advanced (N = 140)	SMD
Age (years)	77 (70 -82)	76 (69–82)	78 (72–83)	0.063	78 (70–83)	77 (71–83)	0.061
Female	70 (19.2)	40 (19.7)	30 (18.6)	0.797	32 (22.9)	27 (19.3)	-0.037
Smoking status	290 (79.7)	166 (81.8)	124 (77.0)	0.263	112 (80.0)	111 (79.3)	-0.068
BMI (kg/m2)	26.1 (23.5–28.7)	26.6 (23.8–29.4)	25.4 (23.3–28.3)	0.014	25.6 (23.4–28.1)	25.7 (23.7–28.5)	0.027
Neoadjuvant chemotherapy	128 (35.2)	83 (40.7)	45 (28.0)	0.010	40 (28.6)	41 (29.3)	0.016
Diabetes mellitus	78 (21.4)	38 (18.6)	40 (24.8)	0.157	31 (22.1)	33 (23.6)	0.083
CCI	6 (4–7)	5 (4–7)	6 (4–7)	0.072	6 (4–7)	6 (4–7)	0.050
ASA ≥ 3	134 (36.8)	60 (29.4)	74 (46.0)	0.001	52 (37.1)	57 (40.7)	0.072
eGFR (ml/min/1,73 m2)	68.0 (50.0–87.0)	71.0 (56.0–89.5)	63.0 (44.0–83.0)	0.001	67.0 (50.0–86.0)	64.5 (46.0–84.3)	-0.060
Hypertension	232 (63.7)	127 (62.3)	105 (65.2)	0.601	92 (65.7)	91 (65.0)	0.015
Congestive heart failure	21 (5.8)	11 (5.4)	10 (6.2)	0.747	10 (7.1)	7 (5.0)	-0.030
Prior surgery	121 (33.2)	71 (34.8)	50 (31.1)	0.431	48 (34.3)	46 (32.9)	-0.077
Prior radiotherapy	19 (5.2)	15 (7.4)	4 (2.5)	0.037	13 (9.3)	4 (2.9)	-0.413
U.D				0.231			
Ileal conduit	271 (74.5)	158 (77.8)	113 (70.2)		111 (79.3)	96 (68.6)	-0.203
Ureterocutaneostomy	35 (9.6)	8 (3.9)	27 (16.8)		8 (5.7)	23 (16.4)	0.249
Neobladder	58 (15.9)	37 (18.2)	21 (13.0)		21 (15.0)	21 (15.0)	0.000

As a check on the assumptions underlying the PSM approach, the degree of overlap in the propensity score distributions between the two groups was evaluated and found to be satisfactory (Supplementary Fig. 2). Additionally, the SMD for the propensity score among matched patients was 0.076, further supporting the validity of the matching process in achieving covariate balance.

### Final pathological characteristics

Patients with locally advanced BCa exhibited higher rates of lymphovascular invasion (92.5% vs 31.5%, p < 0.001) and urethral positive surgical margins (11.8% vs 4.4%, p = 0.009) compared with those with localized disease. The distribution of pathological T stage was consistent with the predefined classification, with 19 patients (11.8%) classified as locally advanced due to the presence of nodal involvement (pT0–2/N1) (Table 2).

**Table 2:** Final pathological characteristics between groups in all and matched populations

median (q1-q3) / n (%)	Overall (N = 364)	Localized (N = 203)	Advanced (N = 161)	p-value	Localized (N = 140)	Advanced (N = 140)	p-value
Urethra positive surgical margins	28 (7.7)	9 (4.4)	19 (11.8)	0.009	8 (5.7)	18 (12.9)	0.039
LVI	213 (58.5)	64 (31.5)	149 (92.5)	< 0.001	45 (32.1)	129 (92.1)	< 0.001
Final pathology							
T0		31 (15.3)			24 (7.9)		
Ta		17 (8.4)			10 (7.1)		
Tis		57 (28.1)			44 (31.4)		
T1		47 (23.2)			28 (20.0)		
T2a		46 (22.7)			32 (22.8)		
T2b		5 (2.5)			2 (1.4)		
T3a			80 (49.7)			72 (51.4)	
T3b			10 (6.2)			10 (7.1)	
T4a			43 (26.7)			35 (25.0)	
T4b			9 (5.6)			8 (5.7)	
T0-2/N1			19 (11.8)			15 (10.7)	

### Perioperative parameters

The median operative time is longer in the advanced BCa group (360 min (IQR 300–400) vs 350 min (IQR 300–390). The localized BCa group showed a median estimated intraoperative blood l.

oss of 550 ml (IQR 350–800), which was less than that of the locally advanced BCa (IQR 700 ml, 500–1100). All other perioperative data regarding wound infection, ileus, pneumonia, and cardiovascular complications were comparable between both groups (Table 3).

**Table 3:** Comparison between perioperative and postoperative outcomes between groups in all and matched populations

median (q1-q3) / n (%)	Overall (N = 364)	Localized (N = 203)	Advanced (N = 161)	p-value	Localized (N = 140)	Advanced (N = 140)	p-value
Post complication	255 (70.1)	132 (65.0)	123 (76.4)	0.018	94 (67.1)	108 (77.1)	0.062
Wound infection	36 (9.9)	20 (9.9)	16 (9.9)	0.978	13 (9.3)	13 (9.3)	1.000
Abdominal	40 (11.0)	23 (11.3)	17 (10.6)	0.815	64 (45.7)	69 (49.3)	0.840
abscess							
Clavien-dindo							
> = 2	172 (47.3)	93 (45.8)	79 (49.1)	0.537	14 (10.0)	13 (9.3)	0.550
0	80 (22.0)	48 (23.6)	32 (19.9)	0.340	33 (23.6)	26 (18.6)	0.317
1	112 (30.8)	62 (30.5)	50 (31.1)		43 (30.7)	45 (32.1)	
2	125 (34.3)	69 (34.0)	56 (34.8)		48 (34.3)	51 (36.4)	
3	37 (10.2)	22 (10.8)	15 (9.3)		15 (10.7)	11 (7.9)	
4	5 (1.4)	1 (0.5)	4 (2.5)		0 (0.0)	3 (2.1)	
5	5 (1.4)	1 (0.5)	4 (2.5)		1 (0.7)	4 (2.9)	
Urinary infection	41 (11.3)	22 (10.8)	19 (11.8)	0.773	16 (11.4)	16 (11.4)	1.000
Sepsis	60 (16.5)	27 (13.3)	33 (20.5)	0.066	17 (12.1)	29 (20.7)	0.053
Urinary leak	27 (7.4)	12 (5.9)	15 (9.3)	0.218	5 (3.6)	13 (9.3)	0.051
Cardiovascular	45 (12.4)	22 (10.8)	23 (14.3)	0.321	18 (12.9)	19 (13.6)	0.860
complications							
Bleeding-	89 (24.5)	41 (20.2)	48 (29.8)	0.034	32 (22.9)	40 (28.6)	0.274
transfusion							
Complication	149 (40.9)	74 (36.5)	75 (46.6)	0.051	54 (38.6)	63 (45.0)	0.276
within 90 days							
Death within 90	8 (2.2)	1 (0.5)	7 (4.3)	0.024	1 (0.7)	6 (4.3)	0.120
days							
Blood loss	600 (400–1000)	550 (350–800)	700 (500–1100)	< 0.001	500 (350–800)	700 (500–1100)	< 0.001
Surgery time	360 (300–400)	350 (300–390)	360 (300–400)	0.349	340 (300–380)	360 (305 -400)	0.056
Length of	10 (8–16)	9 (8–15)	11 (8–17)	0.490	10 (8–15)	11 (8–17)	0.223
hospital stay							
Ileus	254 (69.8)	150 (73.9)	104 (64.6)	0.120	102 (72.9)	88 (62.9)	0.073
Pneumonia	55 (15.1)	28 (13.8)	27 (16.8)	0.379	19 (13.6)	21 (15.0)	0.733

As shown in Tables 4, 5, we found a association between the BCa group and intraoperative blood loss in (Beta: 230.10, 95% CI: 89.86; 370.34) and operative time (Beta: 16.48, 95% CI: 2.63; 30.33), after adjusting for counfonder. This finding was confirmed in the sensitivity analysis conducted on the matched population (Supplementary Table 1a-b).

**Table 4:** Multiple logistic regression models* to investigate the association between groups and peri operative and postoperative outcomes in the all population

	OR Advanced vs Localized	95%CI	p-value
Post complication	1.613	0.996; 2.637	0.053
Wound infection	1.083	0.513; 2.261	0.831
Abdominal abscess	0.968	0.479; 1.922	0.925
Clavien-dindo > = 2	1.147	0.742; 1.774	0.536
Urinary infection	1.171	0.588; 2.319	0.650
Sepsis	1.776	0.996; 3.196	0.053
Urinary leak	2.239	0.970; 5.323	0.061
Cardiovascular	1.056	0.540; 2.058	0.872
complications			
Bleeding-transfusion	1.500	0.902; 2.498	0.118
Complication within 90 days	1.428	0.920; 2.219	0.112
Death within 90 days	-	-	-
Abdominal ascess	0.968	0.479–1.922	0.925
Ileus	0.708	0.440–1.138	0.154
Pneumonia	1.234	0.666–2.285	0.502

^*^Adjusted for: age, sex, smoking status, BMI, Neoadjuvant chemotherapy, GFR, diabetes mellitus and ASA

**Table 5:** Multiple linear regression models* to investigate the association between groups and peri operative and postoperative outcomes in the all population

	Beta Advanced vs Localized	95%CI	p-value
Blood loss	230.10	89.86; 370.34	0.001
Surgery time	16.48	2.63; 30.33	0.020
Length of hospital stay	2.21	-0.55; 4.97	0.117

^*^Adjusted for: age, sex, smoking status, BMI, Neoadjuvant chemotherapy, GFR, diabetes mellitus and ASA

### Postoperative parameters

There were no differences between both groups in 30– and 90-day postoperative complications, and the length of hospital stay was comparable between the two groups. Early postoperative complications were similar in both groups, with sepsis and urinary leak tended to be less in the localized BCa group without reaching significance (Table 3). For postoperative parameters no association with BCa groups were found (Tables 4, 5). This finding was confirmed in the sensitivity analysis conducted on the unmatched population (Supplementary Table 1a-b).

### Cancer-related outcomes

Median follow-up, calculated using the reverse Kaplan–Meier method, was 54.9 months (95% CI 45.9–61.3) for patients with localized BCa and 36.4 months (95% CI 31.9–55.3) for those with locally advanced BCa.

The median OS was 76.9 months (95% CI: 70.7-not estimable (N.E.)) in patients with localized BCa, during which 63 patients (31.0%) died. In contrast, the median in patients with locally advanced BCa was 22.3 months (95% CI: 15.3–34.6), with 77 deaths (47.8%).

The localized BCa group, 36 patients (17.7%) experiencing recurrence compared with 89 events (55.3%) in the locally advanced group. At 60 months, the cumulative incidence of recurrence was 23.3% in patients with localized BCa and 65.3% in those with locally advanced BCa.

Similarly, cancer-specific mortality occurred in 35 patients (17.2%) in the localized group and 48 patients (29.8%) in the locally advanced group. At 60 months, the cumulative incidence of cancer-specific mortality was 12.1% in localized BCa and 39.1% in locally advanced BCa.

**Fig. 1:**
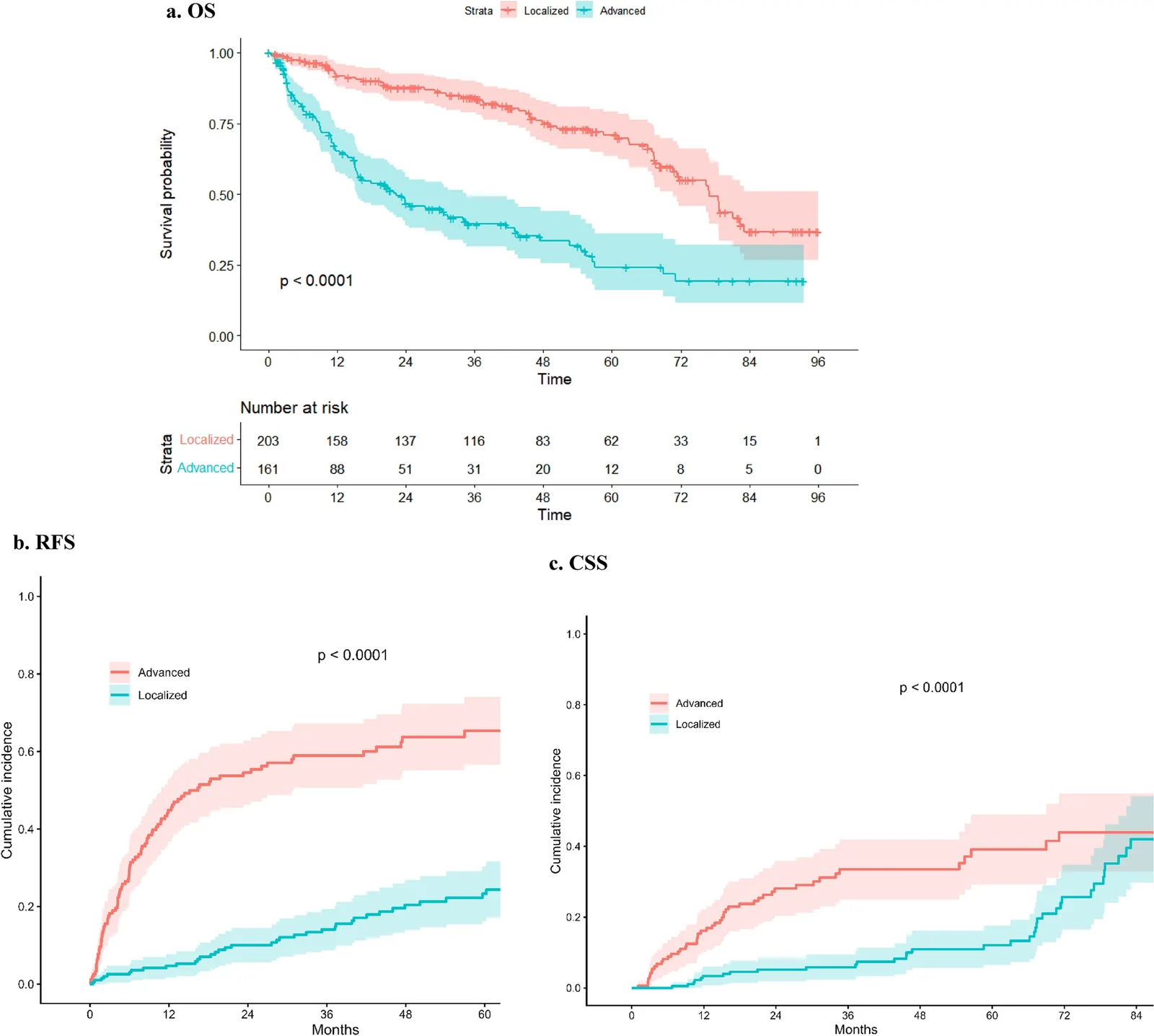
**A** Kaplan–Meier curve for OS with log-rank test. **B** Cumulative incidence curve for RFS. **C** Cumulative incidence curve for CSS. Competing-risk outcomes were compared using Gray’s test

Kaplan–Meier analysis showed significant differences in OS between patients with localized and locally advanced BCa (log-rank p < 0.001). For competing-risk outcomes, cumulative incidence analysis demonstrated significant differences in RFS and CSS between groups (Gray’s test p < 0.001 for both) (Fig. 1).

Survival regression models showed that patients with locally advanced BCa had worse survival outcomes than those with localized BCa. Locally Advanced BCa was associated with an increased risk of overall mortality (OS: HR 3.40, 95% CI 2.40–4.81), higher risk of recurrence (RFS: sHR 5.12, 95% CI 3.45–7.59), and increased cancer-specific mortality (CSS: sHR 2.26, 95% CI 1.43–3.59), (Table 6).

**Table 6:** Multivariable* survival analyses: Cox regression for OS and Fine–Gray competing-risks regression for RFS and CSS

	HR/sHR^a^ Advanced vs Localized	95%CI	p-value
OS	3.397	2.400; 4.806	< 0.001
RFS	5.117	3.452; 7.592	< 0.001
CSS	2.261	1.426; 3.592	0.001

^*^Adjusted for: age, sex, smoking status, BMI, Neoadjuvant chemotherapy, GFR, diabetes mellitus and ASA

^a^HR for OS; sHR for RFS and CSS

All these findings were confirmed in the sensitivity analysis conducted in the matched population (Supplementary Table 2).

## Discussion

The current paradigm for treating muscle-invasive bladder cancer involves radical cystectomy with lymph node dissection, which, while effective, imposes a considerable surgical burden on patients due to its invasiveness. Through ongoing advancements, the surgical method has become more effective in removing the bladder, associated tissues, and adjacent organs in conjunction with lymph node dissection to optimize local and regional disease management [23].

Despite the widespread use of robot-assisted radical cystectomy (RARC) for the treatment of muscle-invasive bladder neoplasms [24], numerous centers also perform the surgery using an open approach. RARC and ORC have not been definitively shown to differ in their postoperative complication rates or oncologic outcomes. In tertiary referral centers, a significant proportion of patients undergoing radical cystectomy harbor extravesical disease (pT3/pT4 or N +), which is a common finding in these specimens [25, 26].

Furthermore, there has been considerable debate in recent years regarding the optimal type of urinary diversion (UD) for patients [27]. Although several articles describe different techniques, none of them have demonstrated a clear advantage, not only in terms of oncological and surgical safety but also in terms of quality of life [28, 29]. This demonstrates that the choice of UD depends entirely on the patient's preference and cancer characteristics.

In this context, our study aims to analyze our experience managing locally advanced bladder neoplasms with an open approach. We used a matching approach to reduce the influence of baseline characteristics on study outcomes, controlling for potential confounders, such as age, BMI, Charlson comorbidity index, prior surgery, or prior radiotherapy.

Regarding complications, Pellegrino et al. provide a strong reminder that postoperative morbidity after robot-assisted radical cystectomy is likely underestimated when complications are not collected prospectively and followed beyond 90 days. In this prospective single-center series, complication and readmission rates were very high, with a substantial proportion of severe events occurring late (including after 3 months), highlighting the limits of standard 30- and 90-day reporting. The study’s main strength is its rigorous methodology (standardized grading, structured follow-up, detailed complication classification), which makes the findings particularly relevant for both clinical counseling and trial design. Overall, the paper’s key message is methodological as much as clinical: better reporting reveals a larger and later complication burden than commonly appreciated [30]. In this comprehensive retrospective study, the locally advanced group showed a marginally higher rate of minor complications, primarily due to increased rates of intraoperative bleeding requiring transfusion. It also notes that patients with locally advanced disease have the most severe complication rates (Clavien Dindo 4–5) even after propensity score-matched analysis (2.1% and 2.9%, respectively). This analysis found no substantial clinical differences in 30-day safety or outcomes for ORC procedures conducted on patients with locally advanced BCa, and this information may be useful for clinicians when counseling patients with clinical T3-T4 or N + disease about the potential benefits and risks of different treatment approaches. Moreover, although the use of minimally invasive techniques, which undoubtedly lead to lower transfusional rates, is not yet widespread in our center, we had low perioperative morbidity and mortality with paralytic intestinal ileus, abdominal abscess, and transfusion among the most common complications [31].

Our analysis indicates that neoadjuvant chemotherapy (NAC) is more commonly administered to patients with localized disease. In contrast, many Patients presenting with locally advanced cT3-T4 tumors also exhibit significant comorbidities or renal failure. This renal impairment often results from large bladder tumors infiltrating the ureteral orifices or from pre-renal failure, ultimately rendering these patients unsuitable for NAC. Additionally, some patients who present to emergency department with large bladder tumors require immediate surgical intervention, such as RC following trans-urethral resection of the bladder tumor (TURBT), due to recurrent episodes of gross hematuria and resulting anemia.

Considering survival outcomes De Angelis et al. show that cisplatin-based neoadjuvant chemotherapy is associated with better survival outcomes in patients with cT2–4aN0M0 bladder cancer treated with radical cystectomy. In the propensity score–matched cohort, NAC was linked to lower 5-year cancer-specific and overall mortality, and this association remained significant in adjusted Cox models, supporting a real-world survival benefit. The survival analysis is a key strength of the paper because it combines matching, multivariable adjustment, and sensitivity analyses, although residual confounding cannot be fully excluded due to the retrospective design [32].

Regarding survival outcomes, we found that the median OS was 78.5 months with 44 (31.4%) deaths in the localized BCa group vs 27.4 months with 77 (55.0%) deaths in the locally advanced BCa group ( *p* < 0.001). The cancer-specific survival was 18.6% and 30.0% for localized and locally advanced BCa (*p* < 0.001). These observations are similar to those already noted in the literature [3, 33]. However, it is important to emphasize that when faced with locally advanced disease, major surgery can be performed in high-volume centers as the postoperative complications are, in any case, comparable to those of localized disease.

While the data presented underscore a shift in the therapeutic landscape, they also raise important questions regarding surgical indications. Given that the results suggest limited oncological benefit, Longoni et al. show that, in non-organ-confined urothelial bladder cancer (T3–4 and/or N1–3), non-surgical management is associated with significantly worse cancer-specific survival than radical cystectomy plus perioperative chemotherapy. After propensity score matching and competing-risk analysis, both bimodal therapy and trimodal therapy had higher 5-year cancer-specific mortality versus RC + CT (bimodal therapy: 66.2% vs 44.9%, mHR 2.12; trimodal therapy: 61.1% vs 46.6%, mHR 1.62; all p < 0.001), with similar results in the T3–4N0 sensitivity analysis. The survival outcome data are the key strength of the paper because they combine PSM with competing-risk regression, but the retrospective SEER design, lack of comorbidity data, and clinical-vs-pathological staging mismatch limit causal interpretation[34].

In accordance Longoni et al. show that, in octogenarians with organ-confined (T2N0M0) urothelial bladder cancer, trimodal therapy is associated with worse cancer-specific survival than radical cystectomy (RC). In this SEER-based cohort, 5-year cancer-specific mortality was markedly higher with trimodal therapy than RC (50.1% vs 31.1%), and this difference remained significant after competing-risk adjustment and after propensity-score matching (HR ~ 1.75), with similar results in the White-only sensitivity analysis. The survival outcomes are the core strength of the paper because they use CSM (not only overall mortality) and competing-risk models in an elderly population where other-cause death is highly relevant[35].

There is evidence of a growing movement towards the centralization of BCa and radical cystectomy to minimize postoperative complications and optimize patient outcomes [36, 37]. Precise and accurate treatment of locally advanced urothelial carcinoma of the bladder is critical in improving patient outcomes and reducing the risk of adverse events and radical cystectomy, an integral component of multimodal therapy for this patient [38, 39]. Patients with pT3 and pT4 BCa should undergo similar surgical treatment, as suggested by Liberman et al., who found a comparable cancer-specific mortality rate, particularly in the absence of lymph node infiltration [26].

Validating preliminary findings is required through future studies involving larger patient cohorts and a standardized follow-up protocol.

Our study has several limitations that should be considered. First, its retrospective design inherently introduces risks of selection bias and residual confounding; therefore, the findings should be interpreted within this context. Nevertheless, retrospective studies such as ours often provide an important basis for future prospective research.

Due to the unique characteristics of our cohort, which were derived from a high-volume tertiary referral center, the incidence of locally advanced bladder cancer (pT3/pT4 or N +) and the clinical scenarios presented may not be representative of other patient populations. In contrast to multicenter studies, where multiple pathologists may be involved, our single-center study had a dedicated uropathologist examine each histological specimen, minimizing interobserver variability [40].

Another limitations of our study is the exclusion of robotic cases. Following the publication of the RAZOR and iROC trials, robotic radical cystectomy has indeed become part of the standard care for these patients [41, 42]. However, in the context of our research, we chose to focus on traditional techniques to maintain sample homogeneity and to avoid confounding variables related to the heterogeneous adoption of robotic technologies during the study period.

Our analysis excluded information on macroscopic residual disease after surgery, as it was not included in the database; only surgeries with curative intent were included. In addition, detailed data on surgical complexity, such as the extent of resection, intraoperative technical difficulty, and other procedure-specific factors, were not systematically recorded, preventing adjustment for their potential impact on perioperative outcomes.

The analysis was restricted to patients with complete data for all variables included in the models. Although the proportion of missing data was limited, this complete-case approach may have reduced the final sample size and introduced potential selection bias.

Importantly, the primary analyses were based on multivariable regression models adjusted for relevant clinical covariates, while propensity score matching was used as a sensitivity analysis to assess the robustness of the findings. However, because disease stage represents an intrinsic biological characteristic rather than a modifiable exposure, neither adjustment nor matching can fully account for confounding related to tumor biology.

Finally, the reliance on observational data introduces the possibility of residual bias, which may not be fully addressed by the matching process.

The findings of this analysis should be replicated in multicenter studies to confirm the results and overcome the limitations inherent to its single-center design. While our analysis provides insight into patients undergoing open RC, it is essential to acknowledge that the findings may not be representative of other departments or surgical approaches.

## Conclusion

In this retrospective matched study, RC for locally advanced bladder cancer is a technically feasible treatment with tolerable therapy and related morbidity and mortality.

Patients should be informed preoperatively about potential postoperative complications and their risk of fatal outcomes, which is essential for obtaining informed consent.

Radical cystectomy for locally advanced BCa can be performed successfully in centers with a high level of expertise. Implementing a centralized model of bladder cancer care enables the integration of multidisciplinary teams, streamlined workflows, and evidence-based practices, ultimately leading to improved patient outcomes and reduced healthcare expenses.

## Conflict of interest

The authors declare no competing interests.

## Data Availability

The data inside the manuscript have been deposited in the ethical committment of our Foundation (Fondazione Agostino Policlinico Gemelli, Rome (ID 2882, approved by the EC on 05/12/2019)).
